# CRISPR–Cas13: Pioneering RNA Editing for Nucleic Acid Therapeutics

**DOI:** 10.34133/bdr.0041

**Published:** 2024-09-06

**Authors:** Guanglin Zhu, Xinzhi Zhou, Mingzhang Wen, Jianjun Qiao, Guo Li, Yuan Yao

**Affiliations:** ^1^ School of Chemical Engineering and Technology, Tianjin University, Tianjin 300072, China.; ^2^ ZJU-Hangzhou Global Scientific and Technological Innovation Center, Zhejiang University, Hangzhou, Zhejiang 311200, China.; ^3^ College of Chemical and Biological Engineering, Zhejiang University, Hangzhou, Zhejiang 310027, China.; ^4^ Zhejiang Institute of Tianjin University, Shaoxing 312300, China.; ^5^ Frontiers Science Center for Synthetic Biology (Ministry of Education), Tianjin University, Tianjin 300072, P. R. China.; ^6^ Xianghu Laboratory, Hangzhou 311231, China.

## Abstract

The CRISPR–Cas13 system has emerged as a revolutionary tool for RNA editing, offering new opportunities for the development of nucleic acid therapeutics. Unlike DNA-targeting CRISPR–Cas9, Cas13 targets and cleaves RNA, enabling gene silencing and preventing genomic instability. Its applications include suppressing disease-causing genes, correcting splicing errors, and modulating immune responses. Despite these advances, challenges persist, such as the need to refine specificity, mitigate off-target impacts, and ensure effective delivery. This review provides an overview of the CRISPR–Cas13 mechanism, elucidating its role in RNA-targeted therapies and its transformative potential for disease treatment. Furthermore, it addresses the ongoing challenges that the scientific community is striving to overcome.

## Introduction

Nucleic acid therapeutics, exemplified by CRISPR technologies, are pioneering innovative approaches for disease treatment [1,2]. The emergence of CRISPR–Cas9-mediated DNA editing has allowed the ability to fix genetic mutations, presenting a potent strategy for disease treatment [3,4]. The CRISPR–Cas13 system has distinguished itself as an effective tool in RNA editing, signifying a notable advancement in the domain of nucleic acid therapeutics [5,6]. The system’s ability to precisely target RNA sequences without inducing genomic instability confers a safety advantage over DNA-targeting systems [7,8].

**Fig. 1.**
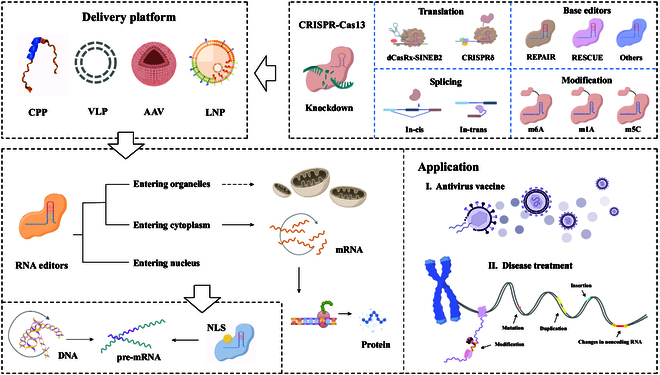
CRISPR–Cas13-based nucleic acid therapeutics.

The CRISPR–Cas13 system, a class 2 type VI system, consists of a single effector, Cas13, that is complexed with CRISPR RNA (crRNA) without the need for tracrRNA [5,6]. The Cas13 protein incorporates nucleotide-binding higher eukaryotes and prokaryotes nucleotide-binding ribonuclease domains enabling it to process precursor crRNA, cleave target RNA, and degrade nonspecific bystander RNA. The Cas13 system, guided by crRNA, stands out for its ability to target RNA sequences without the need for protospacer adjacent motifs, thereby broadening its range of targetability. This feature, coupled with its high efficiency and specificity in RNA manipulation, positions Cas13 as an effective tool for transcriptome engineering. The advent of the CRISPR–Cas13 system has broadened the horizons for RNA editing, introducing innovative tools for RNA therapeutics (Fig. 1). In the field of nucleic acid therapeutics, emphasis is consistently placed on the efficiency, specificity, and delivery mechanisms of these editing tools.

## RNA Knockdown

A variety of Cas13 effectors have been identified, including Cas13a [5,6], Cas13b [9], Cas13c [10], Cas13d [11], Cas13X/Y (also known as Cas13bt) [12,13], and Cas13e to Cas13i [14]. Compared with Cas9, Cas13 offers broader targeting capability for RNA knockdown in mammalian cells. Unlike Cas9, which requires a protospacer adjacent motif for DNA targeting, Cas13 effectors typically show a preference for a specific protospacer flanking sequence (PFS). Notably, RfxCas13d stands out among the Cas13 variants, as it does not require a PFS for targeting and cleaving RNA. This characteristic enhances the versatility and utility of RfxCas13d in RNA editing applications within mammalian systems [11]. RfxCas13d has proven to be highly effective at targeting cellular RNA, achieving greater specificity and higher knockdown rates in both in vitro and in vivo experiments. Its compact molecular size, combined with its high editing efficiency, renders it particularly amenable to delivery via adeno-associated virus (AAV) vectors.

RfxCas13d and sgRNA have been effectively utilized for various therapeutic applications. It has been used to target the *PCSK9* gene, which is instrumental in the treatment of metabolic diseases. By reducing *PCSK9* levels, this approach can help improve conditions related to cholesterol metabolism [15]. The same system has been employed to target the *Tmc1* gene, aiming to prevent autosomal-dominant hearing loss. This result highlights the ability of RfxCas13d to address genetic causes of hearing impairment [16]. In ophthalmology, RfxCas13d has been used to target *VEGFA*, a gene associated with choroidal neovascularization in age-related macular degeneration. By reducing *VEGFA* expression, this strategy has the potential to mitigate the progression of age-related macular degeneration [17]. Furthermore, the system has shown promise in the prevention and treatment of severe acute respiratory syndrome coronavirus 2 infection, suggesting its utility in combating viral diseases [18]. In cancer immunotherapy, RfxCas13d has been used to increase the fitness and antitumor activity of chimeric antigen receptor T cells. Multiplexed transcriptomic regulation and metabolic engineering in primary human T cells have allowed this improvement in the efficacy of chimeric antigen receptor T-cell therapies [19]. These applications underscore the therapeutic potential of RNA-targeting CRISPR systems such as RfxCas13d.

## RNA Base Editing

Over half of human genetic diseases are attributed to single-nucleotide mutations, underscoring the importance of RNA base editing as a means to reverse these point mutations. Cas13-based RNA base editors utilize catalytically inactive CRISPR–Cas13 (dCas13) as the RNA-targeting system and an exogenous deaminase as the effector. When the spacer of the crRNA hybridizes with the target sequence with a single mismatch, the flipped-out nucleotide on the target RNA is captured and catalyzed by deaminase to achieve base alteration at the target site. Two notable advancements in this field are the REPAIR (RNA editing for programmable A-to-I replacement) [20] and RESCUE (RNA editing for specific C-to-U exchange) [21] systems. These platforms achieve A-to-G and C-to-U base conversions, respectively, by fusing deactivated Cas13b with human adenosine deaminase acting on RNA2 (hADAR2) deaminases. As an alternative to h*ADAR2*, a tool called CURE has been developed, which utilizes h*APOBEC3A* fused with dCas13b for precise C-to-U editing [22] (Table 1).

**Table 1.** List of RNA editors.

Tools	Type	Strengths	Limitations
REPAIR	Base editing	• A-to-I	• High transcriptome-wide off-targets by ADAR2
		• No PFS	• Bystander off-targets near targeted adenosine
		• ~40% editing efficiency	• Limited to transcribed sequences
		• No targeting sequence constraints	
		• Independent of endogenous repair pathways	
RESCUE	Base editing	• C-to-U	• High transcriptome-wide off-targets by ADAR2
		• ~40% editing efficiency	• A-to-I off-targets near targeted cytosine
		• Minimal motif preference near target	• Limited to transcribed sequences
		• Independent of endogenous repair pathways	
CURE	Base editing	• C-to-U	• High transcriptome-wide off-target effects
		• High or comparable editing efficiency	• Detectable genomic off-target sites
		• Independent of endogenous repair pathways	• Strict motif preference at target site
CASFx	Alternative splicing	• No PFS	• Confined to cis-sequence manipulation
		• Exon inclusion and exclusion	• Depends on endogenous mRNA splicing
		• Multiplexed splicing	• Limited editing types
		• Comparable editing efficiency	
		• Higher transcriptome-wide specificity	
CRAFT	Trans splicing	• 5′ and 3′ RNA splicing	• Depends on endogenous mRNA splicing
		• Enables various editing types	• Low in vivo splicing efficiency
		• 20% Splicing efficiency in pre-mRNAs	• Complex recombinant CRAFT RNA design
		• Undetectable off-target trans-splicing	• Challenging single AAV delivery Insufficient specificity characterization
Splice Editing	Trans splicing	• 5′ and 3′ RNA editing (simultaneous or separate)	• Depends on endogenous mRNA splicing
		• Insertion/replacement of large RNA segments	• Lower efficiency than CRAFT
		• Enables various editing types	• Requires design of 2 RNAs
		• Comparable efficiency/specificity in GFP reporter	• Difficult single AAV delivery
		• No interference with nontargeted cis-splicing	• Insufficient specificity characterization
RESPLICE	Trans splicing	• 3′ RNA editing	• Depends on endogenous mRNA splicing
		• Enables various editing types	• Slight knockdown of target transcript
		• ~30% Splicing efficiency in endogenous transcripts	• Requires designing 2 RNAs
		• Minimal off-target transcriptome-wide	• Challenging single AAV delivery
			• Low therapeutic efficiency
CRISPR/dCasRx-SINEB2	Translation activation	• Efficient translation activation, +70% for reporter	• Depend on endogenous translation system
		• Minimal off-target effects	
		• No impact on mRNA production/stability	
		• Delivered in single AAV	
		• Induces higher protein levels than traditional tools	
CRISPRδ	Translation repression	• ~80% Efficient translation repression	• Moderate efficiency compared to RNAi
		• Highly specific	
		• No impact on mRNA stability	
		• Suitable for various translation mechanisms	

PFS, protospacer flanking site; RNAi, RNA interference

To increase the precision of RNA base editors, editing tools such as REPAIRv2 [20], REPAIRx [23], RESCUE-S [21], and ecRESCUE [24] have been developed to reduce off-target effects. To accommodate the size limitations of AAV vectors, researchers have utilized smaller Cas13 proteins to create a compact RNA base editor that can be delivered efficiently by a single AAV. Cas13bt1 and Cas13bt3 have been employed to create REPAIR.t1, REPAIR.t3, RESCUE.t1, and RESCUE.t3 editors, respectively [13]. xABE and xCBE editors were engineered by combining dCas13X.1 with the high-fidelity ADAR2dd (deaminase domain of ADAR2) [12]. These innovations exemplify the progress in RNA-targeting CRISPR systems, providing a reliable platform for therapeutic applications.

Cas13-based RNA base editors have been utilized to rectify pathogenic point mutations with high precision and efficacy. For example, a nonsense mutation associated with Duchenne muscular dystrophy can be corrected with mxABE [25]. This approach has demonstrated high editing efficiency, restoring dystrophin expression to levels exceeding 50% of those of the wild type. Furthermore, emxABE has been applied to target the Otoferlin gene, which is implicated in recessive hearing loss. The correction of a missense mutation within the Otoferlin gene has led to a substantial improvement in hearing impairment [26]. These advancements underscore the therapeutic potential of RNA-level genetic correction for a variety of conditions.

## RNA Splicing

Alternative splicing of pre-messenger RNAs (mRNAs) is an essential mechanism of gene expression that, if flawed, can lead to a multitude of human diseases. The deactivated form of Cas13 has provided versatile platforms for targeted correction of aberrant splicing events [27]. dCas13 can be engineered to interact with splicing elements, potentially obstructing the splicing machinery and inducing exon skipping. Moreover, by fusing dCas13 with domains from splicing factors, the precision and efficacy of this intervention can be significantly enhanced, and exon inclusion can also be achieved. CRISPR artificial splicing factor (CASFx) can promote exon inclusion by replacing the RNA recognition motif of splicing factors with dCasRx [28]. The development of inducible systems such as iCASFx, which utilizes the rapamycin-inducible FKBP-FRB dimerization system, provides a means to dynamically control alternative splicing. This chemical-inducible approach allows researchers to modulate splicing events in a controlled and reversible manner. With further advancements in dCas13 technology, the incorporation of serine/arginine-rich (S/R-rich) domains from splicing regulators has yielded a programmable platform termed CASFx-SR. This platform is effective for investigating the roles of splicing factors in tumor biology.

The development of trans-splicing platforms has been driven by the need to manipulate RNA splicing beyond traditional cis-splicing, which occurs within a single RNA molecule. The CRISPR-assisted mRNA fragment trans-splicing (CRAFT) system is an innovative approach that utilizes the Cas13 system to enable the trans-splicing of exogenous RNA into endogenous pre-mRNA [29]. Recombinant CRAFT RNA, which integrates crRNA with splicing elements and exogenous RNA fragments, is a key component of this system. These components are brought into proximity with the donor or receptor sequence by dCas13, facilitating targeted exon modifications. To further increase the recruitment of exogenous RNA fragments, the system can be modified to incorporate the MCP–MS2 hairpin module [30]. This allows dCas13-RBP (RNA-binding protein) fusion to attract exogenous RNA fragments equipped with MS2 hairpin structures, enabling the introduction of large RNA cargoes. RESPLICE is another innovation, as it employs 2 distinct Cas13 effectors [31]. One effector possesses nuclease activity to inhibit cis-splicing, while the other is deactivated to redirect the trans-splicing cargo thereby increasing the overall efficiency of the process. Cas13-mediated trans-splicing is a versatile platform for RNA editing that is capable of inducing a wide array of genetic alterations, including transversions, transitions, insertions, and deletions.

## Epigenetic Modifications in RNA

RNA modification is a pivotal aspect of gene expression regulation, influencing a multitude of biological processes and the progression of various diseases [32]. Among the myriad of posttranscriptional modifications that occur on mammalian mRNA, N6-methyladenosine (m6A) stands out owing to its ubiquity [33]. Harnessing the specificity of the dCas13 system in combination with distinct epigenetic enzymes facilitates the targeted manipulation of these modifications. By fusing dCas13 with either methyltransferases or demethylases, researchers can selectively introduce or remove m6A at specific loci within the nucleus or cytoplasm, thereby minimizing off-target effects [34,35]. The integration of m6A reader proteins, such as YTHDF (YTH domain family protein), with dCas13 further enhances the ability to study the biological functions of specific RNA molecules [36]. Moreover, the combination of the FKBP destabilization domain with dCas13b-ALKBH5, a demethylase, introduces a level of dynamic control over m6A removal [37]. This system can be modulated by the small molecule Shield-1, offering a chemical-inducible approach to regulate m6A in a controlled manner. The m6A writer, a component of the RNA methylation machinery, has been harnessed for anticancer therapy through the fusion of dCas13b with the methyltransferase METTL3. This approach involves introducing m6A modifications onto target tumor suppressor mRNAs, thereby increasing their stability. The strategic enrichment of m6A on specific mRNAs presents a promising avenue for modulating gene expression and combating cancer [38].

The development of a modified m1A eraser, achieved by fusing deactivated Cas13 (dCasRx) with an m1A demethylase, represents a significant advancement in the field of RNA epigenetics. This tool allows for the selective removal of the m1A (1-methyladenosine) modification from specific RNA transcripts. The ability to manipulate m1A levels on RNA precisely enables researchers to explore the relationships between m1A modifications and phenotypic outcomes [39,40]. Furthermore, the reconfigured m5C (5-methylcytosine) modification system offers an approach to control m5C modifications on RNA. By fusing dCasRx with either a methyltransferase or a demethylase, this system provides researchers with the ability to add or remove m5C modifications at specific sites within RNA molecules [41].

## Translation Regulation

Translation regulation is critical for controlling gene expression, and dCas13 offers a unique opportunity to manipulate this process with precision. The platform known as CRISPR/dCasRx-SINEB2 represents an innovative approach to increasing translation efficiency. It does so by coupling the crRNA of dCasRx with the SINEB2 motif, a sequence that is known to increase gene expression by promoting ribosome recruitment. This linkage allows for targeted and efficient translational activation of specific genes while minimizing off-target effects [42]. Conversely, dCas13 can also be employed to inhibit translation. It can achieve this on its own or when combined with a translation repressor, all without causing mRNA degradation. This feature is particularly useful for studying gene function and developing strategies to modulate gene expression. The CRISPRδ platform exemplifies such an application of dCas13 in translational repression. The use of dCas13b obstructs translation initiation in human cells with high specificity because of its robust ability to bind to mRNAs. When dCas13 targets the start codon or the 5’ untranslated region of an mRNA, it prevents ribosome entry or scanning, thereby repressing translation [43].

## Intelligent Design for RNA Editing

The integration of artificial intelligence (AI) with biology has propelled advancements in RNA editing (Table 2). The on-target efficiency and off-target effects of Cas13-mediated RNA editing are influenced by a complex interplay of various factors. These include not only the intrinsic properties of the Cas13 proteins but also the guide RNA sequence, RNA secondary structure, target accessibility, and context dependence, all of which are interrelated in a manner that is both intricate and not fully understood. The advent of AI algorithms and the expansion of screening datasets have provided tools capable of elucidating and predicting the outcomes of RNA editing. For example, targeted inhibition of gene expression via guide RNA design (TIGER) has been trained to forecast the on-target and off-target activities of Cas13d in RNA knockdown based on guide sequence and context information [44]. DeepCas13 is another deep learning model designed to predict on-target efficiency by analyzing features of the sgRNA sequence and RNA secondary structure [45]. Employing this strategy, smaller variants of Cas13 with increased editing efficiency have been successfully developed [46]. The integration of AI technologies enables a more accurate prediction of the efficiency and specificity of Cas13, thereby accelerating the therapeutic deployment of this RNA editing instrument [47,48].

**Table 2.** AI-assisted tool for CRISPR–Cas13

Model	Approach	Cas	Prediction	Features	Link
Cas13design [49]	RF	Cas13d	On-target activity ^a^	Handcrafted guide features; target site context	https://cas13design.nygenome.org
TIGER [44]	CNN	Cas13d	On-target/off-target activity	Guide sequence; target site context	https://tiger.nygenome.org/
DeepCas13 [45]	CNN+RNN	Cas13d	On-target activity	Guide sequences; secondary structure	http://deepcas13.weililab.org
RNAtargeting [47]	CNN	Cas13d	On-target activity	Guide sequence motifs; secondary features	http://RNAtargeting.org
Adapt-seq [50]	CNN	Cas13a	Collateral activity	PFS;mismatches	https://github.com/broadinstitute/adapt-seq-design

RF, random forest; CNN, convolutional neural network; RNN, recurrent neural network

^a^ On-target activity refers to RNA knockdown efficiency.

## Outlook

The advent of CRISPR–Cas13 systems has ushered in a new era of RNA editing, offering a suite of potential therapeutic applications. These systems are particularly valuable for making temporary changes to RNA or for scenarios where DNA editing is challenging. The versatility of CRISPR–Cas13 systems is evident in a range of applications such as diagnostics for RNA viruses, RNA imaging, RNA base editing, RNA epigenome editing, and therapeutic interventions. As researchers move toward clinical applications, ongoing efforts are focused on optimizing RNA editors. Key areas of optimization include enhancing editing efficiency, minimizing RNA off-target effects, and managing the size of the packaging system to facilitate delivery. AI technologies have greatly improved the precision and efficiency of RNA editors by predicting outcomes, including editing efficiencies and off-target effects. This advancement is crucial for reducing unintended genetic modifications, which is especially important in therapeutic applications where safety and efficacy are of utmost importance. In the future, AI methods may be increasingly employed to further personalize RNA editing approaches for therapeutic use.

While the CRISPR–Cas13 system for RNA editing holds great promise, its therapeutic application faces challenges. The potential for collateral cleavage activity means that Cas13-mediated RNA knockdown might introduce unforeseen risks and effects. To address this, a deeper understanding of the mechanism and engineering of Cas13 nucleases is necessary. The size of some Cas13-based tools also presents a challenge for the development of single AAV vector systems. This necessitates the development of more compact Cas proteins or the exploration of alternative strategies such as the recruitment of endogenous effectors. In the clinical setting, the high standards for the efficacy and safety of treatments underscore the importance of increasing the efficiency and specificity of RNA editors through various efforts. Furthermore, the long-term, constitutive expression of Cas13 proteins in vivo raises concerns about potential immunotoxicity and the risk of off-target effects. An increasing number of controllable manipulation methods, such as optogenetics-based and material-based approaches [48–53], are being adapted for Cas13 application. These innovations allow for the controlled expression and tunable activity of Cas13 editing tools, offering the potential for RNA editing applications.

## References

[1] Zhu Y, Zhu L, Wang X, Jin H. RNA-based therapeutics: An overview and prospectus. Cell Death Dis. 2022;13(7):644. 35871216 10.1038/s41419-022-05075-2 PMC9308039

[2] Fellmann C, Gowen BG, Lin PC, Doudna JA, Corn JE. Cornerstones of CRISPR-Cas in drug discovery and therapy. Nat Rev Drug Discov. 2017;16(2):89–100. 28008168 10.1038/nrd.2016.238 PMC5459481

[3] Jinek M, Chylinski K, Fonfara I, Hauer M, Doudna JA, Charpentier E. A programmable dual-RNA-guided DNA endonuclease in adaptive bacterial immunity. Science. 2012;337(6096):816–821. 22745249 10.1126/science.1225829 PMC6286148

[4] Cong L, Ran FA, Cox D, Lin S, Barretto R, Habib N, Hsu PD, Wu X, Jiang W, Marraffini LA, et al. Multiplex genome engineering using CRISPR/Cas systems. Science. 2013;339(6121):819–823. 23287718 10.1126/science.1231143 PMC3795411

[5] Abudayyeh OO, Gootenberg JS, Konermann S, Joung J, Slaymaker IM, Cox DBT, Shmakov S, Makarova KS, Semenova E, Minakhin L. C2c2 is a single-component programmable RNA-guided RNA-targeting CRISPR effector. Science. 2016;353(6299):aaf5573. 27256883 10.1126/science.aaf5573 PMC5127784

[6] Abudayyeh OO, Gootenberg JS, Essletzbichler P, Han S, Joung J, Belanto JJ, Verdine V, Cox DBT, Kellner MJ, Regev A, et al. RNA targeting with CRISPR-Cas13. Nature. 2017;550(7675):280–284. 28976959 10.1038/nature24049 PMC5706658

[7] Adikusuma F, Piltz S, Corbett MA, Turvey M, McColl SR, Helbig KJ, Beard MR, Hughes J, Pomerantz RT, Thomas PQ. Large deletions induced by Cas9 cleavage. Nature. 2018;560(7717):E8–E9. 30089922 10.1038/s41586-018-0380-z

[8] Leibowitz ML, Papathanasiou S, Doerfler PA, Blaine LJ, Sun L, Yao Y, Zhang CZ, Weiss MJ, Pellman D. Chromothripsis as an on-target consequence of CRISPR-Cas9 genome editing. Nat Genet. 2021;53(6):895–905. 33846636 10.1038/s41588-021-00838-7 PMC8192433

[9] Smargon AA, Cox DBT, Pyzocha NK, Zheng K, Slaymaker IM, Gootenberg JS, Abudayyeh OA, Essletzbichler P, Shmakov S, Makarova KS, et al. Cas13b is a type VI-B CRISPR-associated RNA-guided RNase differentially regulated by accessory proteins Csx27 and Csx28. Mol Cell. 2017;65(4):618–630.e7. 28065598 10.1016/j.molcel.2016.12.023 PMC5432119

[10] Yang H, Patel DJ. Structures, mechanisms and applications of RNA-centric CRISPR–Cas13. Nat Chem Biol. 2024;20(6):673–688. 38702571 10.1038/s41589-024-01593-6 PMC11375968

[11] Konermann S, Lotfy P, Brideau NJ, Oki J, Shokhirev MN, Hsu PD. Transcriptome engineering with RNA-targeting type VI-D CRISPR effectors. Cell. 2018;173(3):665–676.e14. 29551272 10.1016/j.cell.2018.02.033 PMC5910255

[12] Xu C, Zhou Y, Xiao Q, He B, Geng G, Wang Z, Cao B, Dong X, Bai W, Wang Y, et al. Programmable RNA editing with compact CRISPR-Cas13 systems from uncultivated microbes. Nat Methods. 2021;18(5):499–506. 33941935 10.1038/s41592-021-01124-4

[13] Kannan S, Altae-Tran H, Jin X, Madigan VJ, Oshiro R, Makarova KS, Koonin EV, Zhang F. Compact RNA editors with small Cas13 proteins. Nat Biotechnol. 2022;40(2):194–197. 34462587 10.1038/s41587-021-01030-2 PMC8929162

[14] Hu Y, Chen Y, Xu J, Wang X, Luo S, Mao B, Zhou Q, Li W. Metagenomic discovery of novel CRISPR-Cas13 systems. Cell Discov. 2022;8(1):107. 36220832 10.1038/s41421-022-00464-5 PMC9554183

[15] He B, Peng W, Huang J, Zhang H, Zhou Y, Yang X, Liu J, Li Z, Xu C, Xue M, et al. Modulation of metabolic functions through Cas13d-mediated gene knockdown in liver. Protein Cell. 2020;11(7):518–524. 32185621 10.1007/s13238-020-00700-2 PMC7095259

[16] Zheng Z, Li G, Cui C, Wang F, Wang X, Xu Z, Guo H, Chen Y, Tang H, Wang D, et al. Preventing autosomal-dominant hearing loss in Bth mice with CRISPR/CasRx-based RNA editing. Signal Transduct Target Ther. 2022;7(1):79. 35283480 10.1038/s41392-022-00893-4 PMC8918553

[17] Zhou C, Hu X, Tang C, Liu W, Wang S, Zhou Y, Zhao Q, Bo Q, Shi L, Sun X, et al. CasRx-mediated RNA targeting prevents choroidal neovascularization in a mouse model of age-related macular degeneration. Natl Sci Rev. 2020;7(5):835–837. 34692105 10.1093/nsr/nwaa033 PMC8288881

[18] Cui Z, Zeng C, Huang F, Yuan F, Yan J, Zhao Y, Zhou Y, Hankey W, Jin VX, Huang J, et al. Cas13d knockdown of lung protease Ctsl prevents and treats SARS-CoV-2 infection. Nat Chem Biol. 2022;18(10):1056–1064. 35879545 10.1038/s41589-022-01094-4 PMC10082993

[19] Tieu V, Sotillo E, Bjelajac JR, Chen C, Malipatlolla M, Guerrero JA, Xu P, Quinn PJ, Fisher C, Klysz D, et al. A versatile CRISPR-Cas13d platform for multiplexed transcriptomic regulation and metabolic engineering in primary human T cells. Cell. 2024;187(5):1278–1295.e20. 38387457 10.1016/j.cell.2024.01.035 PMC10965243

[20] Cox DBT, Gootenberg JS, Abudayyeh OO, Franklin B, Kellner MJ, Joung J, Zhang F. RNA editing with CRISPR-Cas13. Science. 2017;358(6366):1019–1027. 29070703 10.1126/science.aaq0180 PMC5793859

[21] Abudayyeh OO, Gootenberg JS, Franklin B, Koob J, Kellner MJ, Ladha A, Joung J, Kirchgatterer P, Cox DBT, Zhang F. A cytosine deaminase for programmable single-base RNA editing. Science. 2019;365(6451):382–386. 31296651 10.1126/science.aax7063 PMC6956565

[22] Huang X, Lv J, Li Y, Mao S, Li Z, Jing Z, Sun Y, Zhang X, Shen S, Wang X, et al. Programmable C-to-U RNA editing using the human APOBEC3A deaminase. EMBO J. 2020;39(22):e104741. 33058229 10.15252/embj.2020104741 PMC7667879

[23] Liu Y, Mao S, Huang S, Li Y, Chen Y, Di M, Huang X, Lv J, Wang X, Ge J, et al. REPAIRx, a specific yet highly efficient programmable A > I RNA base editor. EMBO J. 2020;39(22):e104748. 33058207 10.15252/embj.2020104748 PMC7667880

[24] Wang Y, Li G, Li X, Wang Y, Huang X, Hu X, Gao J. ecRESCUE: A novel ecDHFR-regulated RESCUE system with reduced RNA off-targeting activity. Cell Commun Signal. 2021;19(1):81. 34332602 10.1186/s12964-021-00759-2 PMC8325194

[25] Li G, Jin M, Li Z, Xiao Q, Lin J, Yang D, Liu Y, Wang X, Xie L, Ying W, et al. Mini-dCas13X-mediated RNA editing restores dystrophin expression in a humanized mouse model of Duchenne muscular dystrophy. J Clin Invest. 2023;133(3):e162809. 36512423 10.1172/JCI162809 PMC9888377

[26] Xue Y, Tao Y, Wang X, Wang X, Shu Y, Liu Y, Kang W, Chen S, Cheng Z, Yan B, et al. RNA base editing therapy cures hearing loss induced by OTOF gene mutation. Mol Ther. 2023;31(12):3520–3530. 37915172 10.1016/j.ymthe.2023.10.019 PMC10727966

[27] Ule J, Blencowe BJ. Alternative splicing regulatory networks: Functions, mechanisms, and evolution. Mol Cell. 2019;76(2):329–345. 31626751 10.1016/j.molcel.2019.09.017

[28] Du M, Jillette N, Zhu JJ, Li S, Cheng AW. CRISPR artificial splicing factors. Nat Commun. 2020;11(1):2973. 32532987 10.1038/s41467-020-16806-4 PMC7293279

[29] Fiflis DN, Rey NA, Venugopal-Lavanya H, Sewell B, Mitchell-Dick A, Clements KN, Milo S, Benkert AR, Rosales A, Fergione S, et al. Repurposing CRISPR-Cas13 systems for robust mRNA trans-splicing. Nat Commun. 2024;15(1):2325. 38485709 10.1038/s41467-024-46172-4 PMC10940283

[30] Borrajo J, Javanmardi K, Griffin J, St Martin SJ, Yao D, Hill K, Blainey PC, Al-Shayeb B. Programmable multi-kilobase RNA editing using CRISPR-mediated trans-splicing. bioRxiv. 2023. 10.1101/2023.08.18.553620

[31] Sita S, Chandrasekaran, Tau C, Nemeth M, Pawluk A, Konermann S, Hsu PD. Rewriting endogenous human transcripts with *trans*-splicing. bioRxiv. 2024. 10.1101/2024.01.29.577779 41653914

[32] Roundtree IA, Evans ME, Pan T, He C. Dynamic RNA modifications in gene expression regulation. Cell. 2017;169(7):1187–1200. 28622506 10.1016/j.cell.2017.05.045 PMC5657247

[33] Boulias K, Greer EL. Biological roles of adenine methylation in RNA. Nat Rev Genet. 2023;24(3):143–160. 36261710 10.1038/s41576-022-00534-0 PMC9974562

[34] Wilson C, Chen PJ, Miao Z, Liu DR. Programmable m6A modification of cellular RNAs with a Cas13-directed methyltransferase. Nat Biotechnol. 2020;38(12):1431–1440. 32601430 10.1038/s41587-020-0572-6 PMC7718427

[35] Xia Z, Tang M, Ma J, Zhang H, Gimple RC, Prager BC, Tang H, Sun C, Liu F, Lin P, et al. Epitranscriptomic editing of the RNA N6-methyladenosine modification by dCasRx conjugated methyltransferase and demethylase. Nucleic Acids Res. 2021;49(13):7361–7374. 34181729 10.1093/nar/gkab517 PMC8287920

[36] Rauch S, He C, Dickinson BC. Targeted m6A reader proteins to study Epitranscriptomic regulation of single RNAs. J Am Chem Soc. 2018;140(38):11974–11981. 30183280 10.1021/jacs.8b05012 PMC6436614

[37] Xu Y, Wang Y, Liang FS. Site-specific m6 a erasing via conditionally stabilized CRISPR-Cas13b editor. Angew Chem Int Ed Engl. 2023;62(43):e202309291. 37713087 10.1002/anie.202309291 PMC10592254

[38] Li A, Cao C, Gan Y, Wang X, Wu T, Zhang Q, Liu Y, Yao L, Zhang Q. ZNF677 suppresses renal cell carcinoma progression through N6-methyladenosine and transcriptional repression of CDKN3. Clin Transl Med. 2022;12(6):e906. 35678231 10.1002/ctm2.906 PMC9178504

[39] Xie S, Jin H, Yang F, Zheng H, Chang Y, Liao Y, Zhang Y, Zhou T, Li Y. Programmable RNA *N*^1^-methyladenosine demethylation by a Cas13d-directed demethylase. Angew Chem Int Ed Engl. 2021;60(36):19592–19597. 34081827 10.1002/anie.202105253

[40] Xie G, Lu Y, He J, Yang X, Zhou J, Yi C, Li J, Li Z, Asadikaram G, Niu H, et al. Small molecule-inducible and photoactivatable cellular RNA N1-methyladenosine editing. Angew Chem Int Ed Engl. 2024;e202320029. 38591694 10.1002/anie.202320029

[41] Zhang T, Zhao F, Li J, Sun X, Zhang X, Wang H, Fan P, Lai L, Li Z, Sui T. Programmable RNA 5-methylcytosine (m5C) modification of cellular RNAs by dCasRx conjugated methyltransferase and demethylase. Nucleic Acids Res. 2024;52(6):2776–2791. 38366553 10.1093/nar/gkae110 PMC11014266

[42] Cao C, Li A, Xu C, Wu B, Liu J, Liu Y. Enhancement of protein translation by CRISPR/dCasRx coupled with SINEB2 repeat of noncoding RNAs. Nucleic Acids Res. 2023;51(6):e33. 36715335 10.1093/nar/gkad010 PMC10085674

[43] Apostolopoulos A, Kawamoto N, Chow SYA, Tsuiji H, Ikeuchi Y, Shichino Y, Iwasaki S. dCas13-mediated translational repression for accurate gene silencing in mammalian cells. Nat Commun. 2024;15(1):2205. 38467613 10.1038/s41467-024-46412-7 PMC10928199

[44] Wessels HH, Stirn A, Méndez-Mancilla A, Kim EJ, Hart SK, Knowles DA, Sanjana NE. Prediction of on-target and off-target activity of CRISPR-Cas13d guide RNAs using deep learning. Nat Biotechnol. 2024;42(4):628–637. 37400521 10.1038/s41587-023-01830-8

[45] Cheng X, Li Z, Shan R, Li Z, Wang S, Zhao W, Zhang H, Chao L, Peng J, Fei T, et al. Modeling CRISPR-Cas13d on-target and off-target effects using machine learning approaches. Nat Commun. 2023;14(1):752. 36765063 10.1038/s41467-023-36316-3 PMC9912244

[46] Zhao F, Zhang T, Sun X, Zhang X, Chen L, Wang H, Li J, Fan P, Lai L, Sui T, et al. A strategy for Cas13 miniaturization based on the structure and AlphaFold. Nat Commun. 2023;14(1):5545. 37684268 10.1038/s41467-023-41320-8 PMC10491665

[47] Wei J, Lotfy P, Faizi K, Baungaard S, Gibson E, Wang E, Slabodkin H, Kinnaman E, Chandrasekaran S, Kitano H, et al. Deep learning and CRISPR-Cas13d ortholog discovery for optimized RNA targeting. Cell Syst. 2023;14(12):1087–1102.e13. 38091991 10.1016/j.cels.2023.11.006

[48] Tong H, Huang J, Xiao Q, et al. High-fidelity Cas13 variants for targeted RNA degradation with minimal collateral effects. Nat Biotechnol. 2023;41(1):108–119. 35953673 10.1038/s41587-022-01419-7

[49] Wessels HH, Méndez-Mancilla A, Guo X, Legut M, Daniloski Z, Sanjana NE. Massively parallel Cas13 screens reveal principles for guide RNA design. Nat Biotechnol. 2020;38(6):722–727. 32518401 10.1038/s41587-020-0456-9 PMC7294996

[50] Metsky HC, Welch NL, Pillai PP, Haradhvala NJ, Rumker L, Mantena S, Zhang YB, Yang DK, Ackerman CM, Weller J, et al. Designing sensitive viral diagnostics with machine learning. Nat Biotechnol. 2022;40(7):1123–1131. 35241837 10.1038/s41587-022-01213-5 PMC9287178

[51] Yu J, Shin J, Yu J, Kim J, Yu D, Heo WD. Programmable RNA base editing with photoactivatable CRISPR-Cas13. Nat Commun. 2024;15(1):673. 38253589 10.1038/s41467-024-44867-2 PMC10803366

[52] Fan N, Bian X, Li M, Chen J, Wu H, Peng Q, Bai H, Cheng W, Kong L, Ding S, et al. Hierarchical self-uncloaking CRISPR-Cas13a-customized RNA nanococoons for spatial-controlled genome editing and precise cancer therapy. Sci Adv. 2022;8(20):eabn7382. 35584220 10.1126/sciadv.abn7382 PMC9116607

[53] Cardiff RAL, Faulkner ID, Beall JG, Carothers JM, Zalatan JG. CRISPR-Cas tools for simultaneous transcription & translation control in bacteria. Nucleic Acids Res. 2024;52(9):5406–5419. 38613390 10.1093/nar/gkae275 PMC11109947

